# Palisading Adenocarcinoma

**DOI:** 10.1007/s12105-024-01671-0

**Published:** 2024-08-21

**Authors:** Melad N. Dababneh, Christopher C. Griffith, Kaitlyn Ooms

**Affiliations:** 1 Pathology and Laboratory Medicine Institute, Cleveland Clinic, 9500 Euclid Avenue L15, Cleveland, OH 44195 USA; 2 Cleveland Clinic Lerner College of Medicine of Case Western Reserve University, Cleveland, OH USA

**Keywords:** Palisading adenocarcinoma, Sublingual gland

## Abstract

Palisading adenocarcinoma is a morphologically distinct salivary gland neoplasm that has been recently described with predilection to the sublingual gland. We report our experience with this neoplasm to corroborate and enrich the literature and further clarify its phenotype.

Palisading adenocarcinoma is a recently described salivary-type neoplasm with dual cell population and neuroendocrine-like appearance, and predilection to the sublingual gland [1]. An institutional search (2008–2023) of sublingual and/or floor of mouth salivary gland tumors yielded five cases with available glass slides. Upon review, one showed similar morphologic and immunophenotypic features to palisading adenocarcinoma.

The patient is a 58-year-old female who initially presented with a swelling of the submental area of one-month duration. Upon examination, a cystic mass of the left sublingual gland was noted. Computed tomography scan revealed a well-demarcated mass measuring 2.5 cm in greatest dimension, and lymphadenopathy was not identified. Histologic sections of the resection specimen show a well-demarcated, thickly encapsulated, multi-lobulated neoplasm with no evidence of peripheral salivary gland/soft tissue, perineural or angiolymphatic invasion (Figs. 1 and 2a). Foci of intratumoral fibrosis are present. The tumor is composed of two cell populations (Fig. 2b); a predominant population of polygonal epithelioid cells with small nucleoli, arranged in trabeculae and pseudo-rosette-like structures (Fig. 2c), and a second population of scattered well-formed ductal structures with occasional mucocytes (Fig. 2d). No high-grade features including marked cytologic atypia, increased mitoses or tumor necrosis are identified.

**Fig. 1:**
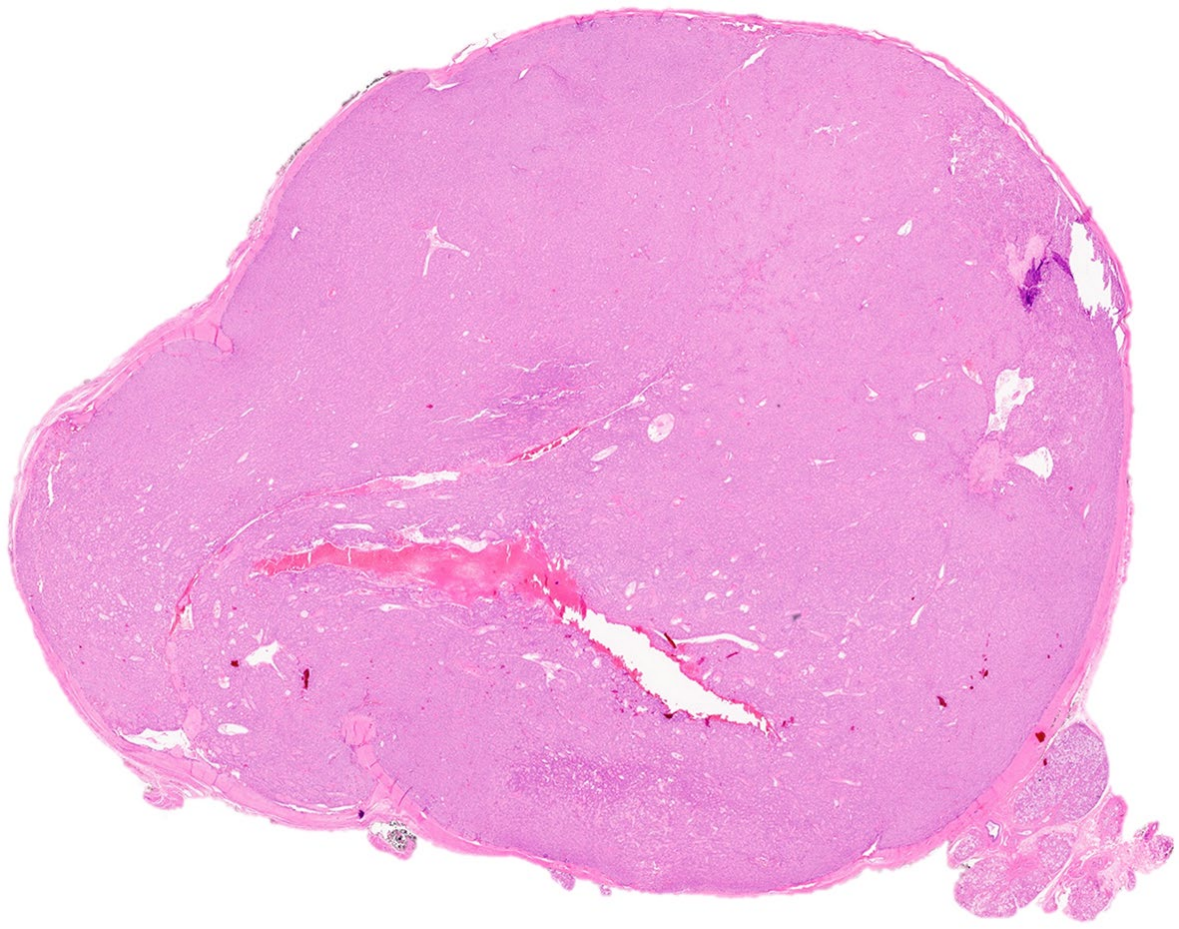
At low power, the tumor is hypercellular, well-circumscribed and encapsulated

**Fig. 2:**
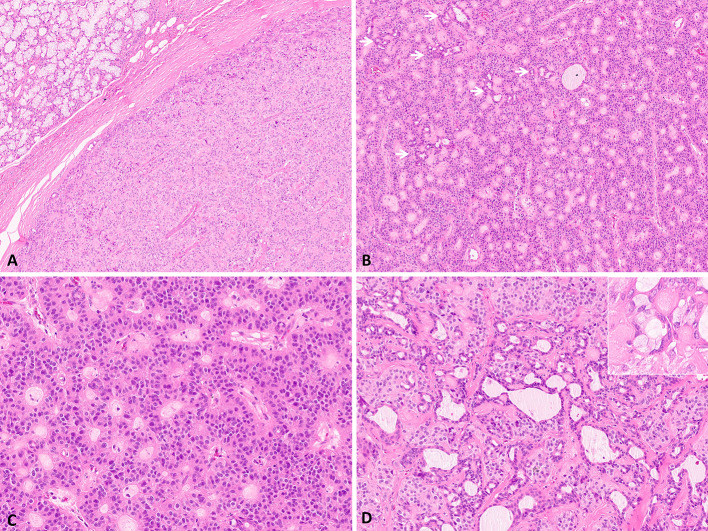
The tumor shows no evidence of invasion into surrounding parenchyma (**a**), and is comprised of two cell components, a predominant palisading component arranged frequently in pseudo-rosettes, and scattered ductal component with eosinophilic cytoplasm and lumens (arrows) (**b**). The predominant cells are polygonal, arranged in trabeculae and pseudo-rosette around hyalinized centers (**c**), while the ductal component is seen frequently in clusters with occasional mucous cells (inset) (**d**)

By immunohistochemistry, the predominant population is positive for CD56 (diffuse), and CAM5.2 (weak), and negative for CK7, CK5/6, synaptophysin, chromogranin, INSM-1, SOX10 and p63. The ductal population is positive for CK7, CK5/6, p63 (abluminal only) and negative-to-equivocal for CD56. S100 is non-specific. SMA and GFAP are negative, and p53 is wild type (Fig. 3).

**Fig. 3:**
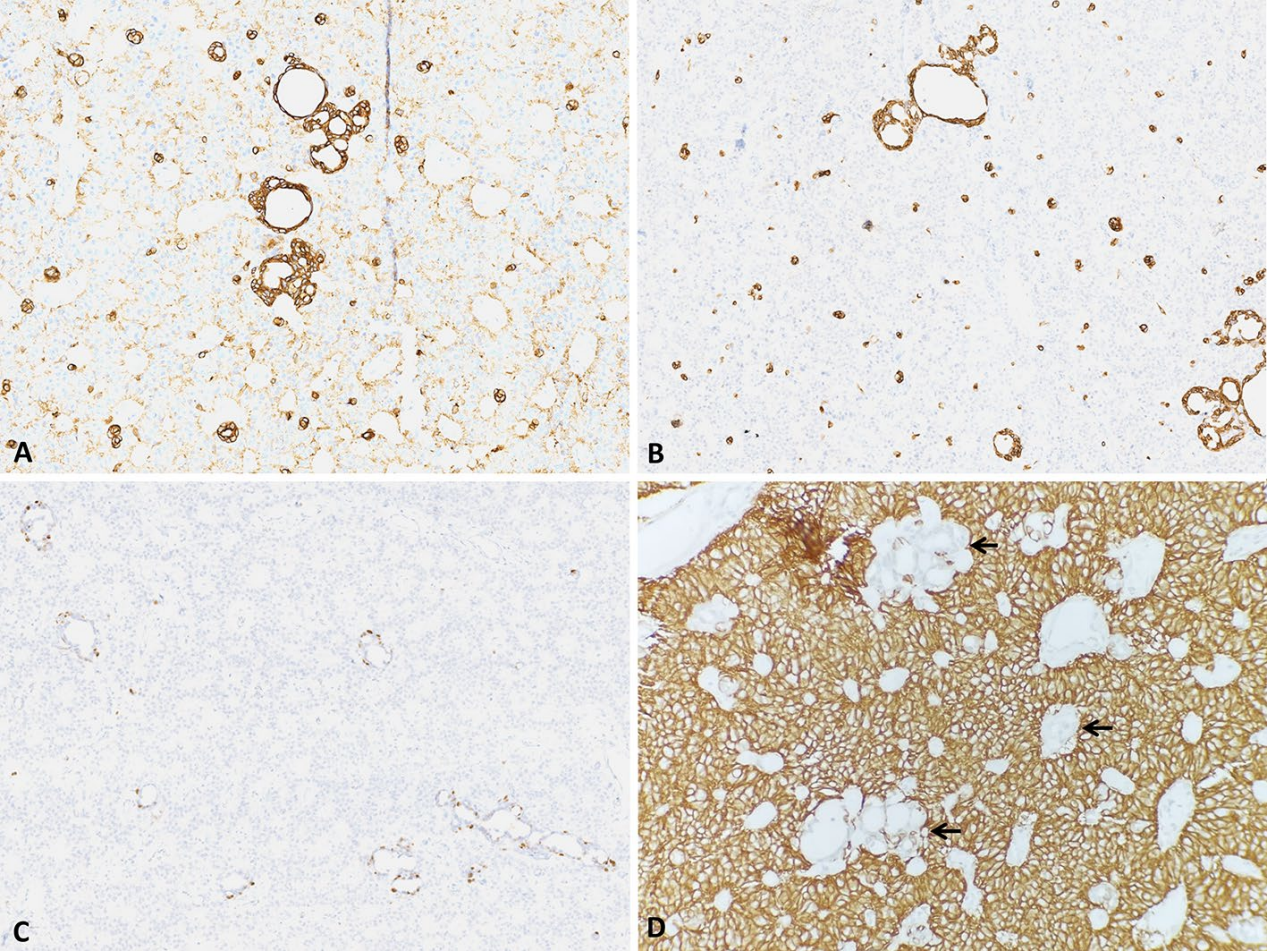
Cam5.2 is strong in ducts and variably positive in the predominant cells (**a**), CK5/6 is strongly positive only in ducts (**b**), P63 is positive in a periductal pattern (**c**), and CD56 is diffusely and strongly positive only in the polygonal cells, while negative-to-equivocal in the ducts (arrow) (**d**)

Electron microscopy was performed at the time, but the images are no longer available for retrieval. Per the report, these palisading cells form structures that have both epithelial and gland-like characteristics but are primitive when compared to the second (apparent ductal) cell population. This is evidenced by a distinct cytoplasmic bipolarity, and formation of small, putative spaces showing short microvillous processes and tight junction attachments characteristic of a gland forming epithelium or neoplasm. No evidence of myoepithelial, neuroepithelial or melanocytic differentiation is identified.

Given the low-grade histomorphology, lack of non-epithelial differentiation and peripheral, lymphovascular or perineural invasion, a diagnosis of “salivary gland adenoma, not otherwise specified” was rendered. There has been no evidence of recurrence or progression post-resection, 15 years later.

In summary, this case shows a gland-forming epithelial neoplasm with dual cell population, similar to the recently described “palisading adenocarcinoma”. The patient is originally from Turkey, supporting a possible geographic association to the eastern hemisphere as is suggested in the initial report [1]. Interestingly, Four of nine cases reported by Bishop et al. [1] and one by Manini et al. [2] showed no evidence of invasion, and combined with our case, 54.5% (6/11) of these neoplasms are theoretically benign or of uncertain biologic behavior. This raises the possible use of “palisading adenoma” terminology in this setting, similar to that used with non-invasive biphasic basal cell neoplasms. The current case and those recently described highlight how our knowledge and classification of salivary gland tumors is continually expanding.

## Data Availability

No datasets were generated or analysed during the current study.
